# Difficult calls to emergency medical dispatch centres – a mixed method study

**DOI:** 10.1186/s12873-025-01343-4

**Published:** 2025-09-08

**Authors:** Inger K. Holmström, Hans Blomberg, Ulrika Winblad, Douglas Spangler

**Affiliations:** 1 School of Health, Care and Social Welfare, Mälardalen University, Postbox 883, Västerås, SE-72123 Sweden; 2 Department of Public Health and Caring Sciences, Uppsala University, Uppsala, Sweden; 3 Uppsala Center for Prehospital Research, Department of Surgical Sciences, Uppsala University, Uppsala, Sweden

**Keywords:** Authentic calls, Difficult calls, Emergency medical dispatch centres, EMS, Telephone nursing, Telephone triage

## Abstract

**Background:**

At emergency medical dispatch centres (EMDCs) telephone triage takes place in three steps: *identifying the event*,* assessing the caller’s need for support*,* and prioritizing the response.* Some calls are considered to be more difficult to handle than others, and decision support systems may in these situations be of limited help. The aim of this study was to describe and characterize difficult calls to EMDCs.

**Methods:**

Retrospective call data from 2022 to 2023 was extracted for Registered Nurse (RN) dispatchers at three EMDCs in Sweden agreeing to participate in this mixed-method study. Categories of difficult calls were identified based on prior research and operationalized as key-word searches in the free text call notes or as indicators based on structured data. A purposeful selection of calls meeting these criteria were extracted, anonymized, and data regarding categories and the phase on the call in which they occurred then coded. A descriptive quantitative analysis was performed, and logistic regression was used to estimate the association between demographics and the likelihood of high-priority ambulance dispatch.

**Results:**

Over the two-year study period, 14 included RNs handled 27,805 calls. Of these, 4888 calls (17.6%) were identified as potentially difficult calls based on free-text notes and structured data, from which 123 calls were selected for further analysis. The median age of callers were 49 years, and 49% were female. Median call duration was 5.6 min, compared to 5.1 min in the full dataset, and 39.5% of calls resulted in a lights and sirens response. Vague or unclear symptoms and psychiatric problems were the most common difficulties. These could occur in all three phases of the calls and in several of the phases in one single call, with a combination of “assessing and prioritizing” being the most common. Male sex was found to be associated with a higher likelihood of receiving an ambulance with high priority.

**Conclusions:**

Difficult calls, mainly with vague or unclear symptoms and psychiatric problems, are common at EMDCs. The reason for the tendency to prioritize young males higher are seen in other contexts but not in truly high priority cases at dispatch and needs further investigation.

**Supplementary Information:**

The online version contains supplementary material available at 10.1186/s12873-025-01343-4.

## Background

Emergency Medical Dispatch Centres (EMDCs) in Sweden receive about 1.2 million calls per year [1]. In the regions under study, these calls are handled by Registered Nurses (RNs) to assure high medical and nursing competency and provide safe and good quality care. This contrasts with much of the country which employ dispatchers with varied education and professional backgrounds in addition to RNs who in some regions back up dispatchers in a secondary triage role. The RNs’ task is to assess the situation / symptoms, prioritize the patients’ need for help and dispatch an ambulance or refer or divert the caller. Their work is often challenging due to a lack of visual cues, limited knowledge of the patient, a high likelihood of acute patient conditions, and time pressure [2–4]. Many factors affect the structure, content, and flow of the conversation: the organization, the RN or dispatcher, the caller, and the interaction between them [5]. RNs are obliged to perform triage, prioritize ambulance resources so that they are used in an optimal way, and treat every caller in a professional and preferably person-centered manner. For callers, it is essential to be listened to and taken seriously. To enable a safe and correct assessment, RNs need to possess high professional competence and effective communication skills [6], in addition to using an appropriate clinical decision support system (CDSS) [7, 8]. Different types of CDSS’ are used internationally to aid telephone triage. They could be either knowledge driven or data driven [9]. In the present study RNs use a CDSS labelled Medical Decision Support Tool (in Swedish: Medicinskt Beslutsstöd with the acronym MBS) which is structured around patient assessment mnemonics commonly employed in pre-hospital care (ABCDE/OPQRST/AMPLE) and follows a layout whereby initial questions focus on ruling out life-threatening conditions, followed by more detailed, symptom-specific questions to collect details about the patient’s condition and medical history [10]. The RNs may, however, steer the communication in a way that fits the caller’s situation, as the CDSS is not script-based.

Telephone triage takes place in three steps: *Identifying the event*,* assessing the caller’s needs*,* prioritizing the response* [7]. Some calls are, however, more difficult to handle than others, and formal CDSS may in some situations be of limited help [8]. A Danish study showed that “unclear problems” were difficult to handle, and predictors of “unclear problems” included age, ethnicity, day of week, and time of day [11]. Calls for psychiatric reasons or regarding older patients were generally more time-consuming than other calls [12]. In an interview study, RNs reported that aggressive callers or calls with language issues were an increasing phenomenon, which prolonged the interview phase, delaying help for the afflicted person and creating stressful situations for RNs [4]. A Danish study showed that in the dispatchers’ views, callers seem to lack knowledge about how to best utilize the emergency number and the medical dispatching process, which might hamper the call and the triage process [13].

Telephone triage at EMDCs is hence often challenging for RNs and some calls may be particularly difficult to handle. Most studies in this area are based on self-reported data, and studies of actual calls are rare but necessary to enable education and interventions. It is also unknown in which of the call phases (identification, assessment, and prioritization [7]), which presents most difficulties. The aim of this study was therefore to describe and characterize difficult calls to EMDC services.

## Methods

### Design

A descriptive mixed-methods study [14] using authentic recorded EMDC calls as data source.

### Sample and setting

Three EMDCs in Mid-Sweden, staffed solely by RNs participated in the study. Together, these EMDCs employ about 75 RNs and serve about 950,000 inhabitants. They operate 365/24/7 and handle around 114,000 calls yearly. All calls are routinely recorded for quality-assurance, follow-up, and clinical development purposes. All regions use a CDSS designed for use by RNs to prioritize calls [10], as described above.

A total of 14 RNs consented to inclusion in the study. Inclusion was somewhat uneven, with seven, five, and two RNs recruited from each region, respectively. The RNs were between 27 and 63 years of age (mean age 45 years) and had worked as RNs between 3 and 30 years (mean time 15.5 years). Nine were females and five were males, and all were native-Swedish speakers. Thirteen had one or more specialist-educations, mostly in prehospital and emergency care.

### Data collection

All nurses at the EMDC’s were informed about the study at work-place meetings by the first author. They were then invited to participate in the study via email. Two reminders were sent out, as well as a reminder in each region’s weekly employee newsletter. The email consisted of an approval to review their calls for research purposes and a questionnaire with demographic questions. For all nurses providing informed consent, data including e.g. audio-files, time-logs and priority, was extracted from the dispatch system database for the years 2022 and 2023, forming the dataset used in this study. The name, address and social security number of the patients were masked for researchers as was the telephone number and name of the caller and the name of the RN handling the call.

First, categories of difficult calls were identified based on interviews performed in a previous study [4]: *calls with communication barriers*,* calls from agitated or rude callers*,* calls about psychiatric illness*,* calls from third parties*,* calls about rare or unclear situations*,* calls with unknown addresses and calls regarding immediate life-threatening conditions.* These categories were then operationalized as key-word searches in the free text call notes or indicators based on structured data (See Table 2 in the results section). As these searches resulted in a number of calls which exceeded the capacity of the research group to review qualitatively and included a number of false positive matches, author DS performed a secondary selection process to identify calls to include for qualitative review. Metadata regarding the calls (including patient demographics, call date and time, calls duration, number of previous calls, RN and study site, difficult call category, and free-text notes made by the RN) were reviewed and calls were purposively selected. This process was performed with the aim of maximizing variation across patient age and gender, obtaining an even distribution across categories of difficult calls and included sites and RNs (where possible including examples from multiple RNs for each category and vice versa), and selecting calls which were highly representative of the intended meaning of each category. This selection process thus skewed the quantitative distribution of patient and call characteristics compared to the full dataset (see Table 1 in the results section) but provided a rich and varied dataset for qualitative analysis. Upon selecting the calls, the audio records were anonymized by removing mentions of personally identifiable information and provided to the research group for further analysis.

### Analysis

First, a descriptive quantitative analysis was performed using medians and proportions as appropriate. 95% confidence intervals were generated using percentiles of 1000 bootstrapped samples. Hierarchical logistic regression was used to identify associations within the data for the full patient population regarding prioritization level while accounting for clustering at the RN level. All quantitative analysis was performed using R v 4.3.2, and the analysis code and results are available as supplement 1. Second, as part of the in-depth qualitative analysis, a matrix was developed based on the phases - *identifying the event*,* assessing the caller’s needs*,* and prioritizing the response-* described by [7]. The calls selected for qualitative review were then listened to repeatedly by the first author and classified into the matrix with regards to which phase(s) the difficulties occurred, and one of the co-authors separately assessed 20 of the calls.

### Ethical considerations

The study was conducted in accordance with the Declaration of Helsinki (2024) and approved by the Swedish Ethical Review Authority (Dnr. 2021-06496-01). RNs were informed about the project orally at work-place meetings, and in writing before they provided their informed consent. They were informed that participation was voluntary, and that they could abstain from participating in any state without giving a reason. Callers were not informed about the study per Ethical Review Authority approval as names, addresses, and social security numbers were masked for researchers.

## Results

Over the two-year study period the RNs handled a total of 27,805 calls. Of these, 4888 (17.6%) were identified as potentially difficult calls based on free-text notes or structured data, and 123 calls of these were included for in-depth analysis. The included RNs had handled a median of 1944 calls each over the two-year study period. There was a substantial variation in the number of included calls per RN, from a minimum of 280 to a maximum of 3960 calls, as some of the included RN were relatively newly hired when the study started, or worked part-time. A median of 9.5 calls per RN were included in the in-depth analysis, ranging between 4 and 14. Demographics of the full population, population identified as meeting at least one of the criteria for being a difficult call, and calls included in the analysis are presented in Table 1.

**Table 1:** Patient demographics and call characteristics

Measure	Full population	Potential difficult calls	Included difficult calls
N	27 805	4888	123
Median age	70 (69–70)	61 (60–62)	49 (38–56)
Percent female	51.3 (50.6–51.8)	50.7 (49 − 2–52.2)	49 (40.5–57.9)
Median call duration (minutes)	5.1 (5.08–5.13)	5.53 (5.43–5.60)	5.6 (5.06–6.15)
Percent lights & sirens response	24.9 (24.4–25.5)	30.7 (29.5–32.0)	39.5 (30.6–47.6)
Percent referred to non-ambulance care	31.7 (31.3–32.3)	28.2 (27.0–29.5)	27.4 (19.3–35.5)

All values are presented as point estimates and 95% confidence intervals

Compared to the full population, the included difficult calls tended to concern younger patients (median age of 49 vs. 70 years) and contained more priority 1 calls (39.5% vs. 24.9%). In other regards the sample reflected the full population. The distribution of calls across categories for both identified and included difficult calls are presented in Table 2. Note that there are more identified categories than number of identified difficult calls, as a single call could be identified as meeting multiple criteria.

**Table 2:** Description of difficult call categories

Category	Definition	Number Identified (*N* = 5652)	Number Included (*N* = 123)
Language barriers	Keywords: Language	354 (6%)	13 (11%)
Communication difficulty	Keywords: Aphasia, difficult to interview	88 (1.5%)	8 (6.5%)
Alcohol / Drugs	Keywords: Alcohol, drugs	105 (1.9%)	9 (7.3%)
Yelling	Keywords: Yelling	381 (6.7%)	14 (11.4%)
Threats / Anger	Keywords: Threatening, angry	113 (2.0%)	6 (4.9%)
Psychiatric / Suicidal	Keywords: Psych, suicide	1029 (18%)	17 (13.8%)
Repeated callers	Number of calls > 50	485 (8.6%)	9 (7.3%)
Third party callers **	Keywords: Third party, not on scene, secondhand	68 (1.2%)	6 (4.9%)
Rare events	Keywords: Electric, hanging, delivery & ongoing, burn	113 (2.0%)	5 (4.0%)
Vague / unclear symtpoms	Keywords: Vague symptoms, unclear	2202 (39.0%)	12 (9.8%)
Uncertain truthfulness*	Keywords: Explain	103 (1.8%)	4 (3.2%)
Immediate life threats	Priority 1 A (highest priority)	521 (9.2%)	18 (14.6%)
Immediate life threats in children	Priority 1 A (highest priority) & age < 13	90 (1.6%)	2 (1.6%)

*Expression typically used by the call takers in the context of RNs noting that callers’ explanations are suspect and incoherent or does not add up with previous calls made during a short time period, e.g. describing other symptoms and accidents, or addresses

** “Third party callers” include both members of the public who are on scene, and any caller (e.g., family/relatives) who are not on scene

The shortest calls were from other healthcare providers about patients they wanted to hand over to the EMDC, and from angry and intoxicated callers. The longest calls were about psychiatric symptoms in combination with alcohol / drug consumption, supporting CPR while waiting for the ambulance to arrive, rare and unclear conditions, calls with language barriers, and an aggressive patient waiting for police assistance.

The difficulties could occur in all three phases of the call: *identifying the event*,* assessing the caller’s need for support*,* and prioritizing the response*, and in several of the phases in one single call, see Table 3.

**Table 3:** Number of calls presenting difficulties in phase/s

Phase /s	Number of calls presenting difficulties
Identifying the event	9
Assessing the caller’s need for support	34
Prioritizing the event	12
Combination of identifying, assessing and prioritizing	10
Combination of assessing and prioritizing	37
None of the phases (immediate life-threat)	21

The most common phases which included difficulties were hence a combination of assessing and prioritizing, followed by solely assessing. Many of the difficult calls included several complexities, which could present as a combination of for instance language barriers, vague and unclear symptoms and intoxication of alcohol / drugs which made it hard to uncover the severity of the callers’ problems, and the level of care needed. The 21 calls, which did not include any difficulties - except the apparent life-threat - included cardiac arrest, ongoing suicide attempts or severe allergic reactions. These calls were classified as difficult based on the emotional strain as described by RNs [8], rather than presenting with difficulties in the three phases.

Below, two examples of calls presenting several difficulties are presented. Personal characteristics in the calls have been changed to provide confidentiality:

*Example 1; including language barriers*,* a third-party caller and vague and unclear symptoms which generated difficulties in identifying the event and assessing the need for support*

*Caller: Ambulance*,* I want an ambulance…my neighbor*,* she has been ill for two weeks…*

*RN: What has she done?*

*C: She was ill for two weeks…and now she is really ill*,* she cannot breathe or so*,* you know it’s very hard…she only takes a breath every 20 min or so and her heart is beating hard…*

*RN: Has she had any previous illnesses?*

*C: She was ill last week…*

*RN: What kind of illness did she have last week?*

*C: She was ill for one hour; she saw the doctor yesterday…*

*RN: Did she see a doctor yesterday? What did he say?*

*C: Very ill… but she should just stay at home and wait.*

*RN: But when you say very ill*,* what do you mean? Does she have a fever*,* is she vomiting*,* is she coughing…*

*C: One could say she has a fever… and she is vomiting*,* and she hasn’t peed for three days*

*RN: And what did the doctor say?*

*C: I don’t know*,* I didn’t accompany her*,* very ill…*

*RN: OK*,* are you with your neighbor now?*

*C: Yes*,* it is my neighbor*

In this example, it is difficult for the RN to get exact answers to her questions. The nature and urgency-level of the patient’s symptoms is hard to determine. The caller keeps saying “very ill”, but seems to be exaggerating, like when she says, “she only takes a breath every 20 minutes”, “one could say she has a fever” and “hasn’t peed for three days”. The lack of exact answers seems to depend on language barriers and the fact that the caller does not know the neighbor very well. The RNs voice sounds stressed and even more frustrated as the call continues. This call takes just over seven minutes, and ends with the RN sending an ambulance, although not with lights and sirens.

*Example 2; including life threat*,* psychiatric / suicidal caller and vague and unclear symptoms which generated difficulties in assessing the caller’s need for support*,* and prioritizing the response*

*Caller (C): I’m…I’m…jumping in front of train…*

*RN: Are you suicidal – intended to jump in front of a train?*

*C: Well…not suicidal – I missed the train*,* it just passed…*

*RN: Where are you now*,* are you okay?*

*C: I missed the train*,* walking along the railway now…took 45 painkillers’ instead.*

*RN: Oh*,* I see. Please tell me more*,* what has happened today?*

*C: I went to the loo…it smelled so bad*,* I went outside…wanted to jump in front of the train…*

*RN: Okay*,* we need you to get to the hospital as you’ve taken so many painkillers.*

*C: The psychiatric clinic*,* I cannot go there…so many people and so much smelling…*

*RN: I think I need to send someone to help you*,* where are you now?*

*C: I’m not sure – I went along the railway; don’t know how far I’ve gone now…*

In this call, the caller is in a state of distress but still speaks with a neutral voice. The caller seems suicidal, but at the same time denying this. The RN talks with a tender voice and calmly tries to make the caller explain the situation and what kind of help is desired. As the call continued, the RN’s voice became higher in pitch, and she seemed frustrated when not getting any clear answers and did not know the location of the caller. This call took eight and a half minutes and ended when the location was finally established, and transportation to hospital was organized.

### Analysis of prioritization with respect to patient sex and age

Upon qualitative analysis, a tendency to take the issues of male callers more seriously was discerned, motivating a formal quantitative analysis. To assess the association between prioritization decisions and the patients’ sex and age, logistic regression models were fit to the data concerning the full population of patients (*n* = 27 805), finding a weak but statistically significant association between male sex and a higher likelihood of receiving an ambulance with a high priority (OR 1.077 (1.019–1.138) vs. females) (Table 4). No association between sex and the likelihood of receiving an ambulance overall was identified, though the coefficient was in the same direction. With regards to age, a significant association was identified with respect to the likelihood of receiving any ambulance response (OR 1.584 (1.544–1.626) for a one standard deviation difference in age, or 26 years), but no association with the likelihood of receiving a priority 1 response was identified.

**Table 4:** Logistic regression coefficients

	Priority 1 dispatch	Any ambulance dispatch
Male sex	1.077 (1.019–1.138)	1.042 (0.989–1.098)
Age (scaled)	1.011 (0.984–1.040)	1.584 (1.544–1.626)

Model coefficients reported as Odds Ratios with 95% confidence intervals. Age is scaled such that the coefficient represents the odds ratio for a one standard deviation difference in age (26 years)

## Discussion

This study aimed to describe and characterize difficult calls to EMDC-services. Out of 27,805 calls, 17.6% were identified as potentially difficult as described by [4]. This shows that handling EMDC calls is a complex and demanding task. The most common difficulties concerned vague and unclear symptoms and psychiatric illness /suicide. This is in line with Møller et al.’s [11] study in Denmark. Third party calls and communication problems like aphasia were the least common difficulties in the present data. Such issues and non-normative symptom descriptions are related to under-triage of symptoms, according to [15]. In the present study, however, the calls resulted in a higher proportion of lights and sirens ambulance responses, compared to the full population. This might be attributed to the application of the “better safe than sorry”- principle applied by RNs in cases of ambiguity and uncertainty [8].

It is interesting to note that RNs in a previous study described that agitated and rude callers, as well as language problems from non-native callers as most challenging [4]. These issues were, however, not the most common type of difficult calls in the present data. This does not contradict the fact that they might be perceived as hard to handle. Eriksson et al. [16] showed in a study of difficult non-emergency calls, that when RNs felt emotional concern, the calls were perceived as hard to handle. RN- patient communication is often emotionally laden, especially in emergency situations, and negative emotion impacts the communication [15]. Feelings of inadequacy, uncertainty, and anxiety evoked among RNs in difficult calls have previously been described as common [16]. If RNs take on a non-professional role or communicate in a non-professional way, the decision-making might be hampered [15]. This was apparent also in the present study. When communication issues arose, regardless of non-native speakers or callers with aphasia, the call-process was hampered as it became difficult to identify the event, assess the need for support and subsequently prioritize the response. In these cases, the CDSS seemed to be of limited help, as a clear symptom needs to be identified to proceed in the system. Spangler et al. [17] showed that increased compliance with CDSS has the potential to improve patient safety. Given the magnitude of difficulties, and complexity in all phases of the calls, RNs need robust support from management and organization in handling these calls in a constructive and patient safe way. This could be provided through mentoring by senior staff, stimulated recall sessions [18] with feedback and training in communication skills. Gerwing et al. [19] reported positive results of a communication training intervention to improve alignment between dispatcher sand callers, which generated behavioral change among dispatchers. Furthermore, reliable CDSS’ are an aid available for RNs [10], and assessment and priority-setting in difficult calls could be aided by video live-streaming. This is implemented in the studied organizations, but there is no strict guidance on when to use it except when the call takers find it suitable. Generally, however, it is still not routinely used in Swedish EMDC settings, but tests show promising results [20, 21]. The RNs also need education and training to handle callers who are vague, have communication difficulties or are intoxicated. Educational interventions at the EMDCs may improve the handling of difficult calls [11]. These could be both theoretical on communication strategies and assessment of unclear and difficult cases, and in workshops with role-playing or stimulated recall sessions [18].

Difficulties in EMDC calls may also be due to a misalignment between the organizational goals and caller /public expectations [22], which was also described by [11]. In the present study this seemed common, as many of the calls included callers’ demanding an ambulance but had a hard time describing why, and the nature of the illness / accident as in example 2 in the result section. A description of someone being “very ill” is indeed hard to assess for RNs, as previously described by [15]. Gerwing & Indseth [23] concluded that while callers’ ability to articulate a problem or symptom contributes directly to dispatchers’ picture of what is happening, the dispatcher him- or herself plays a crucial role in ensuring mutual understanding.

Regarding differences in assessment in relation to callers’ gender, there was an association between male sex and a higher likelihood of receiving an ambulance with a high priority. This is in line with earlier research in other telephone nursing contexts. Kaminsky et al. [24] found that when fathers called, they twice as often as mothers received a doctor’s appointment for their child, while mothers instead were given self-care advice. This could not be explained by differences in the severity of the children’s symptoms [24]. The same pattern was also found in calls made by adult callers for themselves [25]. However, newer studies from a Dutch setting showed that male and female callers with chest discomfort received similar high urgency allocation [26], and that ambulances were dispatched equally to males and females with acute coronary syndrome [27]. Regarding age differences in outcomes of calls, a Danish study found that undertriage tended to be more likely for calls concerning patients over 60 years compared with younger callers in high-risk calls [28]. This issue hence needs further exploration, as the Swedish Health Care Act (2017:30) stipulates good and equal care to all citizens regardless of e.g. age and gender.

### Strengths and limitations

A strength of the study includes the large database of authentic EMDC calls, and furthermore that the categories in Table 2 were derived empirically. However, the list of keywords may not be comprehensive, and some might have been missed/omitted, which is a potential limitation. The Swedish setting could be a potential limitation, as guidelines for call-handling might differ between contexts and settings. Furthermore, the in-depth analysis is limited to 123 calls, and 14 out of 75 RNs agreed to participate in the present study. Timmis et al. [29] pointed out that achieving adequate response rates to surveys in nursing research is a challenge, and that this underrepresentation might introduce potential bias in research. This might hold true also in the present study. Furthermore, the calls selected were not random but rather were selected to capture as broad a set of patient, dispatcher and call characteristics as possible, and it cannot be ruled out that the selection process impacted the findings. An important limitation on the quantitative analysis with regards to age and sex is that the identified associations could be due to either differences in how male and female patients are assessed by RNs, or in differences in the underlying acuity of the conditions for which males and females contact the EMCD.

## Conclusions

In total, 17.6% of calls to the three EMDCs in this study included one or more difficulties, with vague or unclear symptoms and psychiatric problems as the most common in the 123 calls selected for in-depth analysis. These difficulties could occur in all three steps of the call - *identifying the event*,* assessing the caller’s need for support*,* and prioritizing the response* – with a combination of difficulties in assessing and prioritizing being the most common. There was a tendency for male callers and callers of younger age to have a higher prioritization of their calls, which needs to be further investigated. RNs handling EMDC calls should be provided with more training and support to manage difficult calls.

## Supplementary Information

Below is the link to the electronic supplementary material.

Supplementary Material 1

## Data Availability

The datasets generated and/or analysed in this study are not publicly available due to the highly sensitive nature of individual health status data as per the decision by the Ethical Review Board.
