# Supplementary material for: Difficult calls to emergency medical dispatch centres – a mixed method study
Source: BMC Emerg Med. 2025 Sep 8;25:179. doi: 10.1186/s12873-025-01343-4 (PMC12418652; doi:10.1186/s12873-025-01343-4)
Supplement: Supplementary file 1 — Supplementary Material 1 [file 12873_2025_1343_MOESM1_ESM.pdf]

Supplement 1: Difficult calls to emergency medical dispatch centres – A mixed method study

Contents

Distribution by region 2
Distribution by priority 3
Distribution by RN 4
Distribution by gender 5
Distribution by age 6
Distribution by call duration 8
Distribution by category 11
Distribution by gender and priority 12
Distribution of priority by included RN 13
Analysis of priority by gender and age 14

This document contains all code and output used to generate the quantitative results reported in the study: Difficult calls to emergency medical dispatch centres – A mixed method study.

```
library(tidyverse)
```

```
## -- Attaching core tidyverse packages ------------------------ tidyverse 2.0.0 --
## v dplyr     1.1.4     v readr     2.1.5
## v forcats   1.0.0     v stringr   1.5.1
## v ggplot2   3.5.1     v tibble    3.2.1
## v lubridate 1.9.3     v tidyr     1.3.1
## v purrr     1.0.2
## -- Conflicts ------------------------------------------ tidyverse_conflicts() --
## x dplyr::filter() masks stats::filter()
## x dplyr::lag()    masks stats::lag()
## i Use the conflicted package (<http://conflicted.r-lib.org/>) to force all conflicts to become errors
```

```
library(knitr)
library(readxl)
library(lme4)
```

```
## Loading required package: Matrix
##
## Attaching package: 'Matrix'
##
## The following objects are masked from 'package:tidyr':
##
##     expand, pack, unpack
```

```
ci_bootstrap <- function(x,
                         fun,
                         conf_level = 0.95,
                         n_bootstrap = 1000) {
  boot_medians <- replicate(n_bootstrap, {
    sample_x <- sample(x,
```

```
                        length(x),
                        replace = TRUE)
    fun(sample_x)
  })
  lower_bound <- quantile(boot_medians,
                           (1 - conf_level) / 2)
  upper_bound <- quantile(boot_medians,
                           1 - (1 - conf_level) / 2)
  return(c(lower_bound, upper_bound))
}

prios <- c("1"="1A","2"="1B","3"="2A","4"="2B","5"="3",
           "6"="Referral","7"="Referral","47"="Referral")
```

```
data_diff <- data_full %>%
  mutate(across(starts_with("c"),as.numeric)) %>%
  mutate(sum = rowSums(select(.,starts_with("c")))) %>%
  filter(sum>0)
```

```
data_incl <- read_excel("241003_svara_samtal_full.xlsx") %>%
  mutate(prio_group = factor(case_when(pout %in% c("1A","1B") ~ "Prio 1",
                                       pout %in% c("2A","2B") ~ "Prio 2",
                                       .default = "Referral"),
                             levels = c("Prio 1",
                                        "Prio 2",
                                        "Referral"))) %>%
  mutate(DispatchUser_grouped = plyr::revalue(as.character(FK_DispatchUser),
                                              user_map))
```

```
## The following `from` values were not present in `x`: 457, 666, 662
```

Distribution by region

All data

```
table(data_full$region)
```

```
##
##    Sörmland     Uppsala Västmanland
##        4563       13923        9319
```

Potential difficult calls

```
table(data_diff$region)
```

```
##
##    Sörmland     Uppsala Västmanland
##         903        2129        1856
```

Included difficult calls

```
table(data_incl$region)
```

```
##
##    Sörmland     Uppsala Västmanland
```

```
##          20          55          49
```

Distribution by priority

All data

```r
table(data_full$prio_group)
```

```
##
##   Prio 1   Prio 2 Referral
##     6936    12035     8834
```

```r
prop.table(table(data_full$prio_group))*100
```

```
##
##   Prio 1   Prio 2 Referral
## 24.94515 43.28358 31.77126
```

```r
ci_bootstrap(data_full$prio_group == "Prio 1",fun=mean)
```

```
##      2.5%     97.5%
## 0.2442340 0.2547752
```

```r
ci_bootstrap(data_full$prio_group == "Referral",fun=mean)
```

```
##      2.5%     97.5%
## 0.3124258 0.3234679
```

Potential difficult calls

```r
table(data_diff$prio_group)
```

```
##
##   Prio 1   Prio 2 Referral
##     1501     2008     1379
```

```r
prop.table(table(data_diff$prio_group))*100
```

```
##
##   Prio 1   Prio 2 Referral
## 30.70786 41.08020 28.21195
```

```r
ci_bootstrap(data_diff$prio_group == "Prio 1",fun=mean)
```

```
##      2.5%     97.5%
## 0.2939802 0.3193535
```

```r
ci_bootstrap(data_diff$prio_group == "Referral",fun=mean)
```

```
##      2.5%     97.5%
## 0.2694354 0.2941950
```

Included difficult calls

```r
table(data_incl$prio_group)
```

```
##
##   Prio 1   Prio 2 Referral
##       49       41       34
```

```r
prop.table(table(data_incl$prio_group))*100
```

```
##
##   Prio 1   Prio 2 Referral
## 39.51613 33.06452 27.41935
```

```r
ci_bootstrap(data_incl$prio_group == "Prio 1",fun=mean)
```

```
##      2.5%     97.5%
## 0.3145161 0.4758065
```

```r
ci_bootstrap(data_incl$prio_group == "Referral",fun=mean)
```

```
##      2.5%     97.5%
## 0.2016129 0.3467742
```

Distribution by RN

All data

```r
data_full %>%
  group_by(DispatchUser_grouped) %>%
  summarise(n = n()) %>%
  pull(n) %>%
  summary()
```

```
##    Min. 1st Qu.  Median    Mean 3rd Qu.    Max.
##     280    1080    1944    1986    2730    3960
```

```r
table(data_full$DispatchUser_grouped)
```

```
##
##   95  111  188  240  261  331  351  401  453  467  534  567  661  665
##  624  976 2466 3598  280 2749 3960 2672 1421  322 2510 3429 1406 1392
```

Potential difficult calls

```r
data_diff %>%
  group_by(DispatchUser_grouped) %>%
  summarise(n = n()) %>%
  pull(n) %>%
  summary()
```

```
##    Min. 1st Qu.  Median    Mean 3rd Qu.    Max.
##    44.0   221.2   343.5   349.1   467.2   756.0
```

```r
table(data_diff$DispatchUser_grouped)
```

```
##
##  95 111 188 240 261 331 351 401 453 467 534 567 661 665
## 127 214 407 432  44 490 621 347 243  56 479 756 332 340
```

Included difficult calls

```r
data_incl %>%
  group_by(DispatchUser_grouped) %>%
  summarise(n = n()) %>%
```

```
  pull(n) %>%
  summary()
```

```
##    Min. 1st Qu.  Median    Mean 3rd Qu.    Max.
##   4.000   6.500   9.500   8.857  10.750  14.000
```

```
table(data_incl$DispatchUser_grouped)
```

```
##
## 111 188 240 261 331 351 401 453 467 534 567 661 665  95
##  10   9  11   5   9  14  12   6   5   8  11  10  10   4
```

Distribution by gender

All data

```
kon_all <- table(data_full$kon)

prop.table(kon_all)
```

```
##
##         K         M
## 0.5125697 0.4874303
```

```
ci_bootstrap(data_full$kon == "K",fun=mean)
```

```
##      2.5%     97.5%
## 0.5066346 0.5182539
```

Potential difficult calls

```
kon_diff <- table(data_diff$kon)

prop.table(kon_diff)
```

```
##
##         K         M
## 0.5065466 0.4934534
```

```
ci_bootstrap(data_diff$kon == "K",fun=mean)
```

```
##      2.5%     97.5%
## 0.4932385 0.5196450
```

Included difficult calls

```
kon_incl <- table(data_incl$kon)

prop.table(kon_incl)
```

```
##
##         K         M
## 0.4919355 0.5080645
```

```
ci_bootstrap(data_incl$kon == "K",fun=mean)
```

```
##      2.5%     97.5%
```

```
## 0.4112903 0.5887097
```

Distribution by age

All data

```r
hist(data_full$age)
```

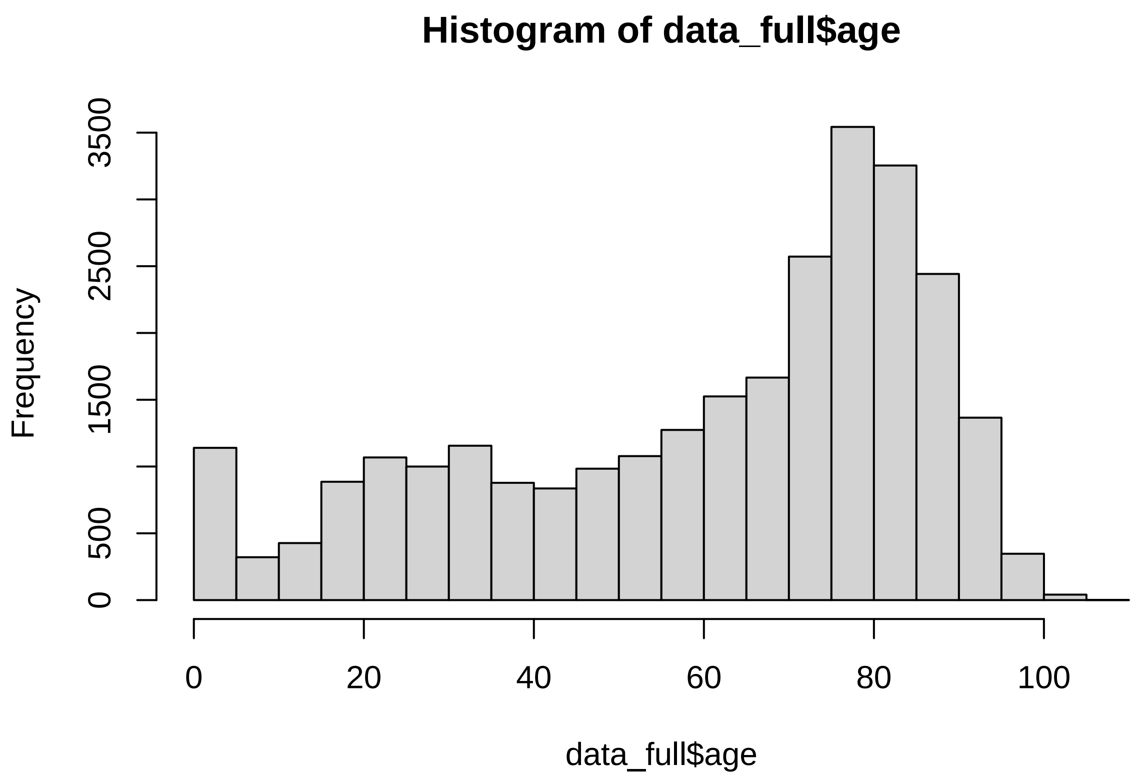


```r
summary(data_full$age)
```

```
##    Min. 1st Qu.  Median    Mean 3rd Qu.    Max.
##    0.00   41.00   70.00   60.72   81.00  106.00
```

```r
ci_bootstrap(data_full$age,fun=median)
```

```
##  2.5% 97.5%
##    69    70
```

Potential difficult calls

```r
hist(data_diff$age)
```

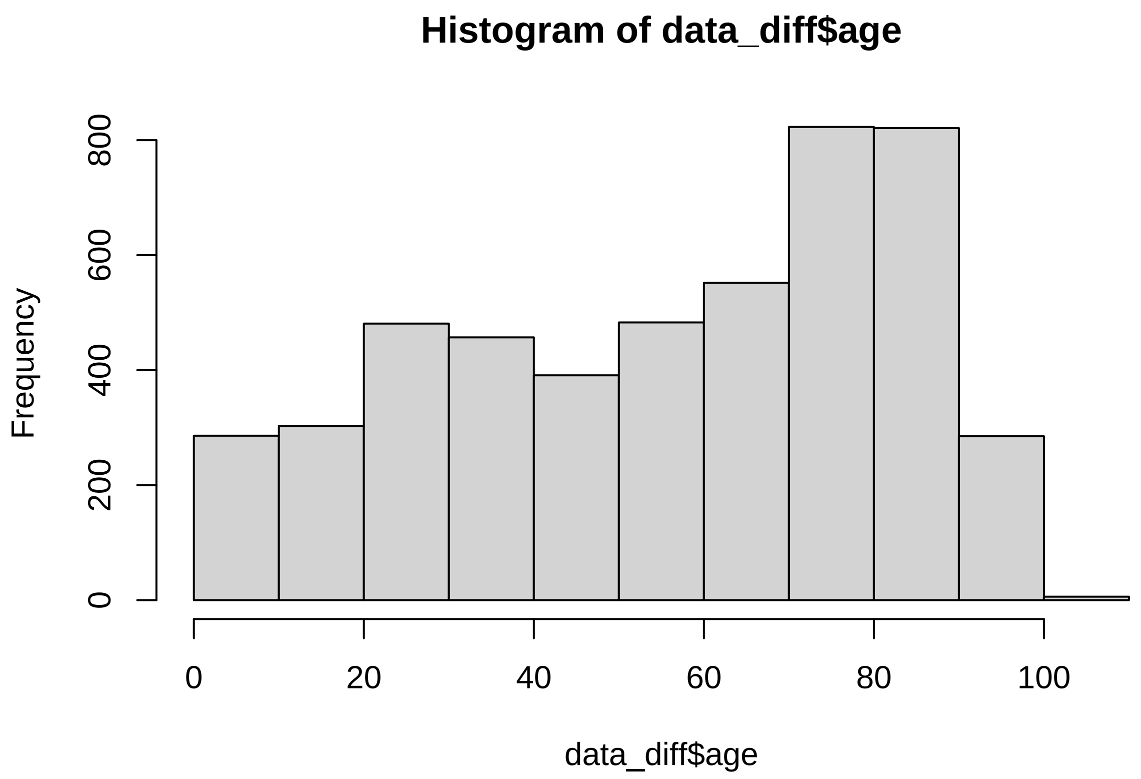


```r
summary(data_diff$age)
```

```
##    Min. 1st Qu.  Median    Mean 3rd Qu.    Max.
##     0.0    33.0    61.0    56.2    79.0   104.0
```

```r
ci_bootstrap(data_diff$age,fun=median)
```

```
##  2.5% 97.5%
##    60    63
```

Included difficult calls

```r
hist(data_incl$age)
```

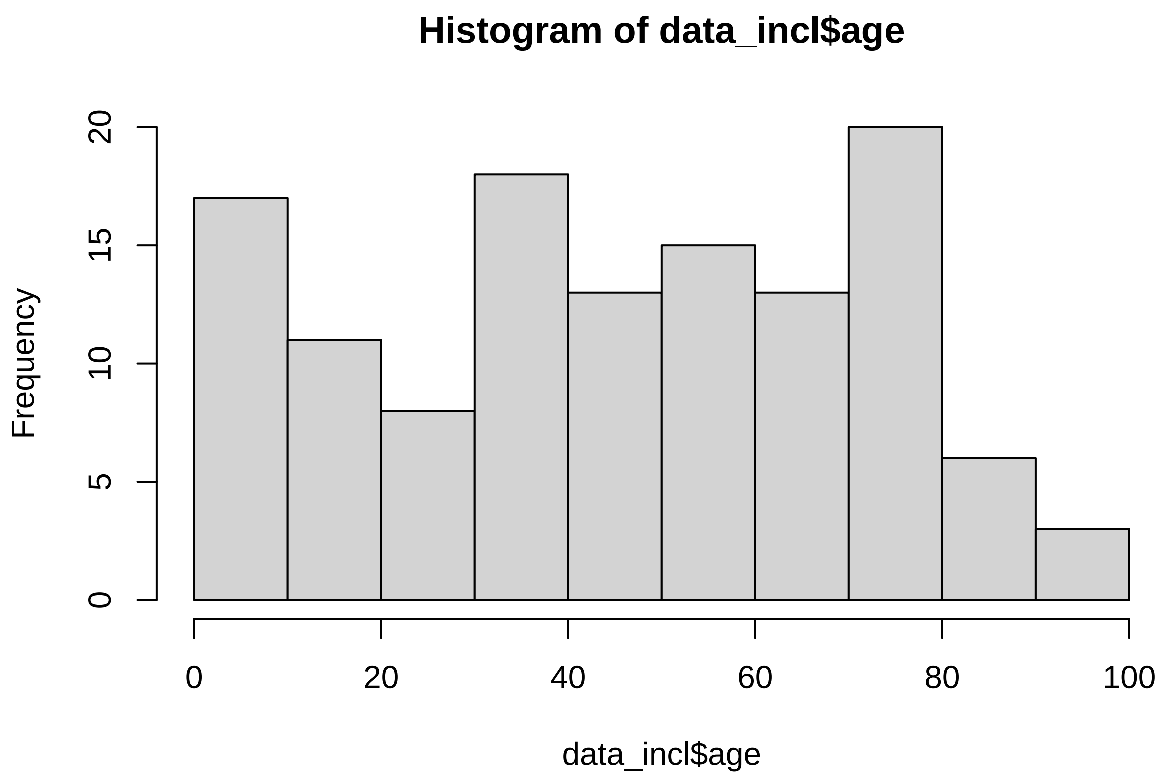


```
summary(data_incl$age)
```

```
##    Min. 1st Qu.  Median    Mean 3rd Qu.    Max.
##    0.00   25.75   49.50   45.98   67.00   93.00
```

```
ci_bootstrap(data_incl$age,fun=median)
```

```
##    2.5%   97.5%
## 38.4875 56.0000
```

Distribution by call duration

All data

```
hist(data_full$duration)
```

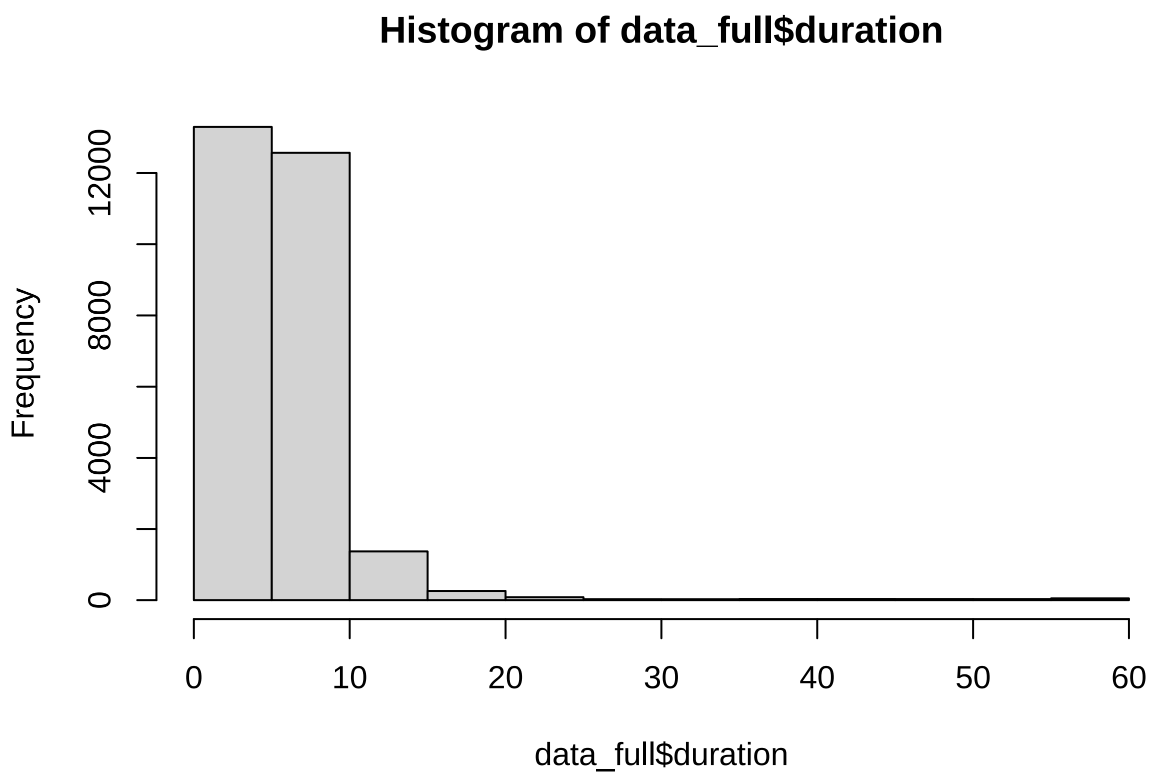


```r
summary(data_full$duration)
```

```
##    Min. 1st Qu.  Median    Mean 3rd Qu.    Max.
##   3.000   4.027   5.111   6.087   6.756  59.916
```

```r
ci_bootstrap(data_full$duration,fun=median)
```

```
##     2.5%    97.5%
## 5.086555 5.140241
```

Potential difficult calls

```r
hist(data_diff$duration)
```

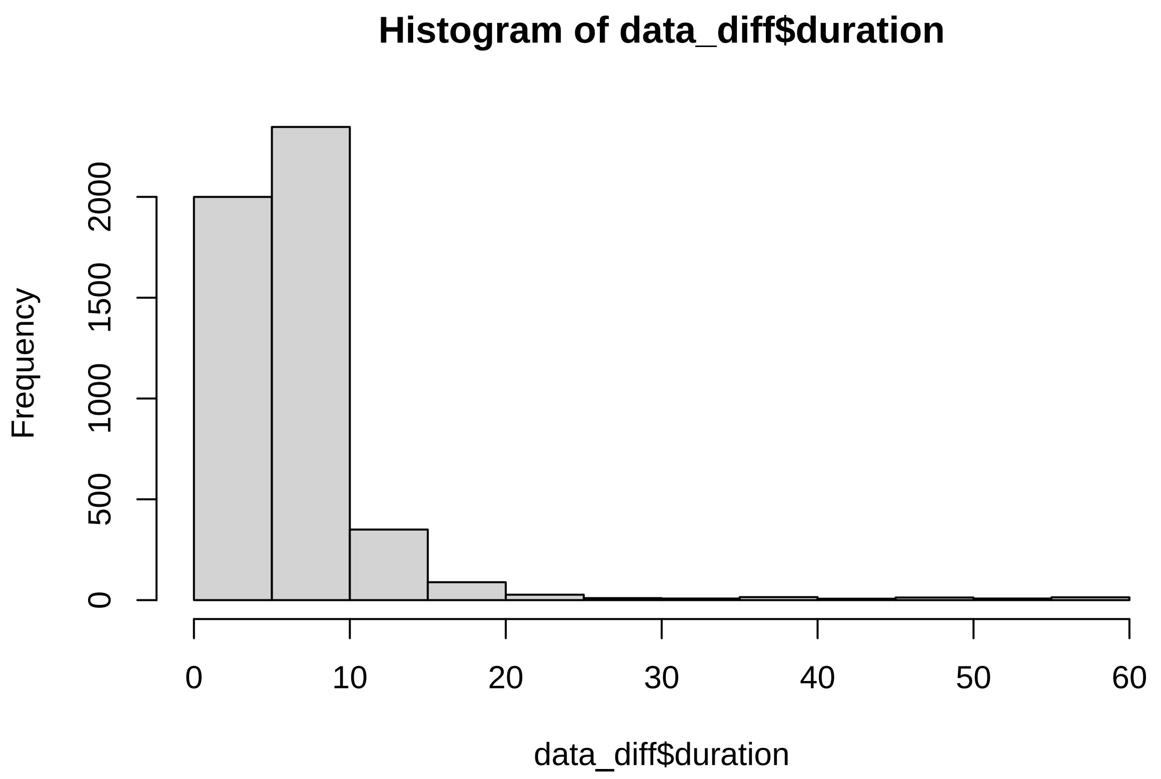


```
summary(data_diff$duration)
```

```
##    Min. 1st Qu.  Median    Mean 3rd Qu.    Max.
##   3.001   4.232   5.531   6.852   7.584  59.813
```

```
ci_bootstrap(data_diff$duration,fun=median)
```

```
##     2.5%    97.5%
## 5.438195 5.605022
```

Included difficult calls

```
hist(data_incl$duration)
```

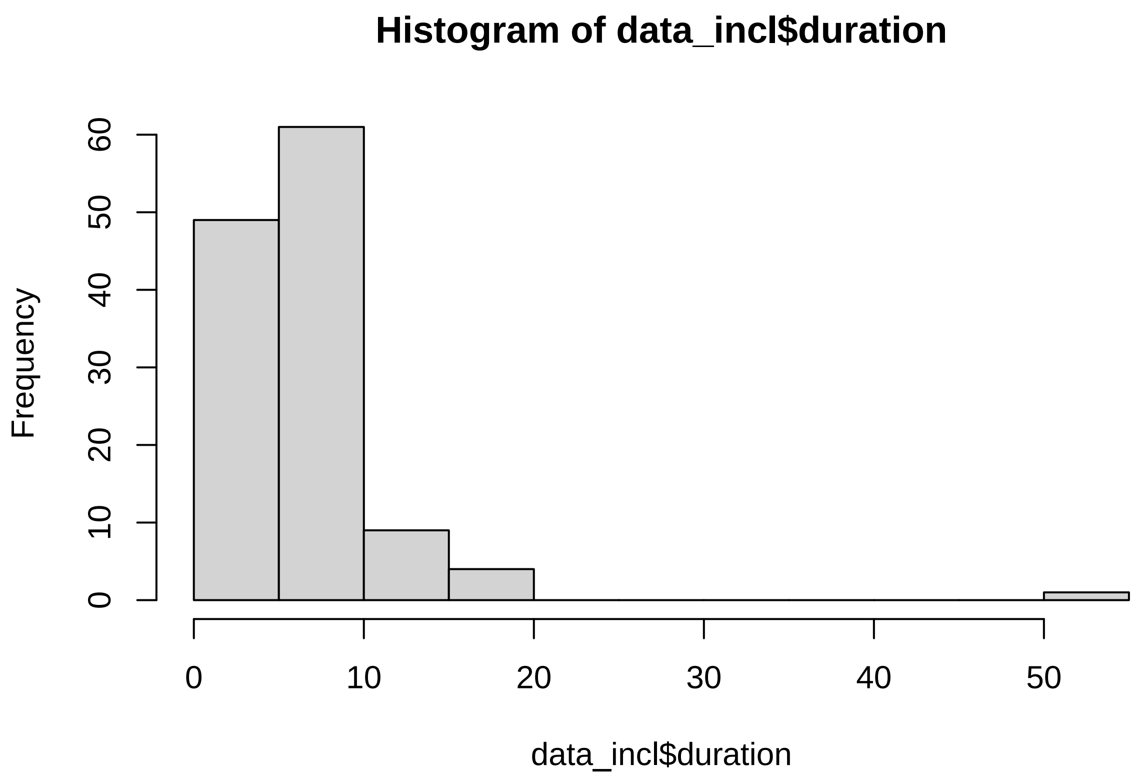


```
summary(data_incl$duration)
```

```
##    Min. 1st Qu.  Median    Mean 3rd Qu.    Max.
##   3.076   4.392   5.664   6.759   7.254  50.410
```

```
ci_bootstrap(data_incl$duration,fun=median)
```

```
##     2.5%    97.5%
## 5.059774 6.142148
```

Distribution by category

All data

```
data_full %>%
  pivot_longer(cols = starts_with("c")) %>%
  group_by(name) %>%
  summarise(n = sum(value)) %>%
  kable()
```

name	n
c01_sprak	354
c02_afasi	88
c03_droger	105
c05_skrik	381
c06_hot	113
c07_psyk	1029

name	n
c08__mang	485
c10__tredjepart	68
c11__sallsynt	113
c12__oklar	2202
c13__ljuger	103
c15__livshot	521
c16__barnlivshot	90

Included difficult calls

```
data_incl %>%
  group_by(name) %>%
  summarise(n = n()) %>%
  kable()
```

name	n
c00__ingen	1
c01__sprak	13
c02__afasi	8
c03__droger	9
c05__skrik	14
c06__hot	6
c07__psyk	17
c08__mang	9
c10__tredjepart	6
c11__sallsynt	5
c12__oklar	12
c13__ljuger	4
c15__livshot	18
c16__barnlivshot	2

Distribution by gender and priority

All data

```
table(data_full$pout,data_full$kon)
```

```
##
##                K    M
##    1A        206  315
##    1B       3267 3148
##    2A       5061 4705
##    2B       1174 1095
##    3         391  474
##    Referral 4153 3816
```

```
prop.table(table(data_full$pout,data_full$kon),margin = 2)
```

```
##
##                      K          M
##    1A       0.01445411 0.02324209
```

```
## 1B       0.22923099 0.23227330
## 2A       0.35510806 0.34715561
## 2B       0.08237440 0.08079392
## 3        0.02743475 0.03497381
## Referral 0.29139770 0.28156128
```

Potential difficult calls

```r
table(data_diff$pout,data_diff$kon)
```

```
##
##              K   M
## 1A         206 315
## 1B         531 449
## 2A         868 777
## 2B         184 179
## 3           26  27
## Referral   661 665
```

```r
prop.table(table(data_diff$pout,data_diff$kon),margin = 2)
```

```
##
##                    K          M
## 1A       0.08319871 0.13059701
## 1B       0.21445880 0.18615257
## 2A       0.35056543 0.32213930
## 2B       0.07431341 0.07421227
## 3        0.01050081 0.01119403
## Referral 0.26696284 0.27570481
```

Included difficult calls

```r
table(data_incl$pout,data_incl$kon)
```

```
##
##               K  M
## 1A            7 18
## 1B           12 12
## 2A           18 14
## 2B            2  7
## Hänvisning   22 12
```

```r
prop.table(table(data_incl$pout,data_incl$kon),margin = 2)
```

```
##
##                      K          M
## 1A         0.11475410 0.28571429
## 1B         0.19672131 0.19047619
## 2A         0.29508197 0.22222222
## 2B         0.03278689 0.11111111
## Hänvisning 0.36065574 0.19047619
```

Distribution of priority by included RN

```r
data_full %>%
  group_by(DispatchUser_grouped,prio_group) %>%
  mutate(DispatchUser_grouped = as.factor(DispatchUser_grouped)) %>%
  summarise(n = n()) %>%
  ggplot(aes(fill=prio_group, y=n, x=DispatchUser_grouped)) +
    geom_bar(position="fill", stat="identity")
```

```
## `summarise()` has grouped output by 'DispatchUser_grouped'. You can override
## using the `.groups` argument.
```

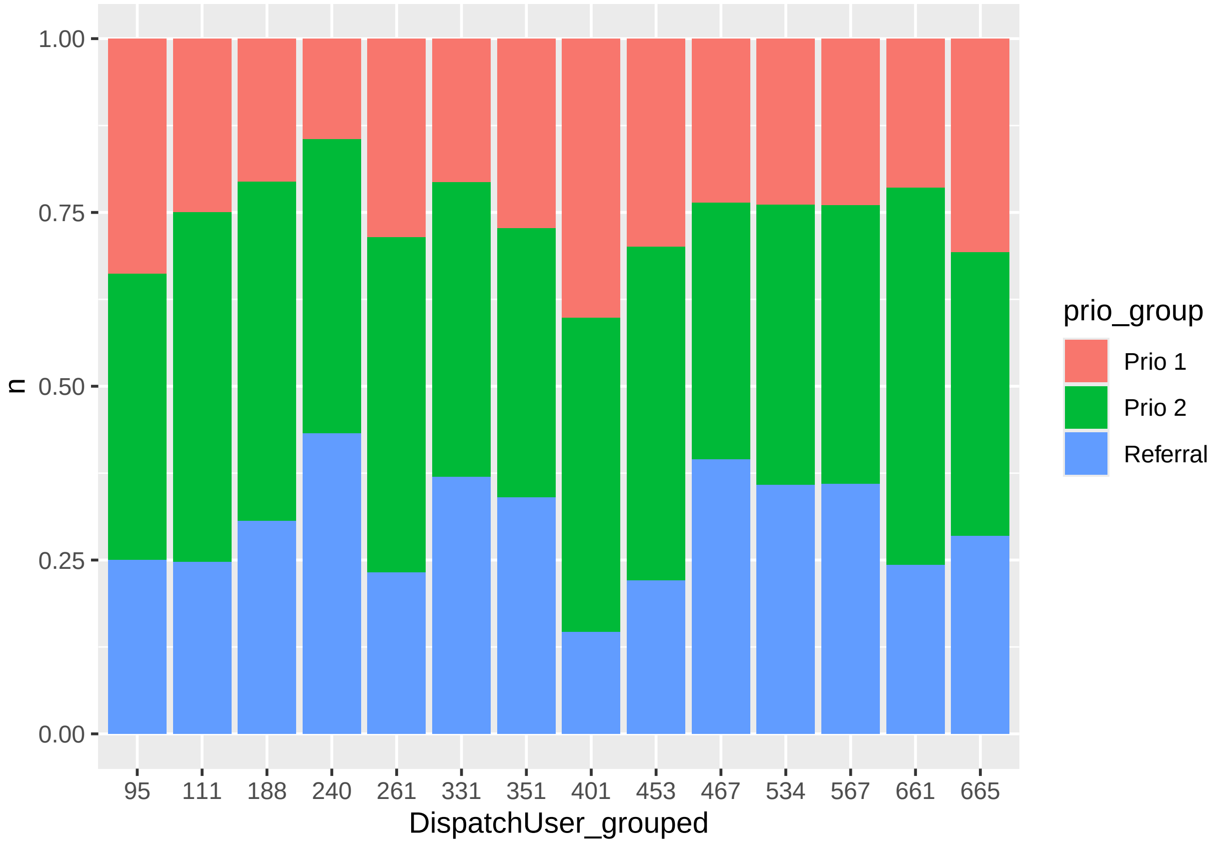

Analysis of priority by gender and age

Descriptive analysis of absolute and proportional distributions

```r
tt <- table(data_full$pout,data_full$kon)
tt
```

```
##
##               K    M
##   1A        206  315
##   1B       3267 3148
##   2A       5061 4705
##   2B       1174 1095
##   3         391  474
##   Referral 4153 3816
```

```r
prop.table(tt,margin = 2)
```

```
##
##                    K          M
##   1A       0.01445411 0.02324209
##   1B       0.22923099 0.23227330
##   2A       0.35510806 0.34715561
##   2B       0.08237440 0.08079392
##   3        0.02743475 0.03497381
##   Referral 0.29139770 0.28156128
```

```r
prop.test(tt)
```

```
##
##  6-sample test for equality of proportions without continuity correction
##
## data:  tt
## X-squared = 45.411, df = 5, p-value = 1.197e-08
## alternative hypothesis: two.sided
## sample estimates:
##    prop 1    prop 2    prop 3    prop 4    prop 5    prop 6
## 0.3953935 0.5092751 0.5182265 0.5174086 0.4520231 0.5211444
```

Fit Ordinal and logistic regression models

```r
data_full$prio <- ordered(data_full$pout,
                          levels = c("Referral", "2B", "2A", "1B", "1A"))

data_full$prio_group <- ordered(data_full$prio_group,
                          levels = c("Referral", "Prio 2", "Prio 1"))

fit.olr <- MASS::polr(prio ~ kon + age,
                      data = data_full,
                      Hess = T)

summary(fit.olr)
```

```
## Call:
## MASS::polr(formula = prio ~ kon + age, data = data_full, Hess = T)
##
## Coefficients:
##        Value Std. Error t value
## konM 0.07059  0.0221762   3.183
## age  0.01057  0.0004407  23.994
##
## Intercepts:
##              Value   Std. Error t value
## Referral|2B -0.1873  0.0320    -5.8558
## 2B|2A        0.2022  0.0321     6.2916
## 2A|1B        1.7711  0.0338    52.4083
## 1B|1A        4.6384  0.0541    85.8022
##
## Residual Deviance: 72393.07
## AIC: 72405.07
```

```
## (865 observations deleted due to missingness)
```

```
fit.olr.grp <- MASS::polr(prio_group ~ kon + age,
                          data = data_full,
                          Hess = T)

fit.glm.p1 <- glmer(I(prio_group == "Prio 1")
                    ~ kon + scale(age) + (1|UserName),
                      data = data_full,
                   family = "binomial")

fit.glm.p12 <- glmer(I(prio_group %in% c("Prio 1","Prio 2"))
                     ~ kon + scale(age) + (1|UserName),
                      data = data_full,
                   family = "binomial")

summary(fit.olr.grp)
```

```
## Call:
## MASS::polr(formula = prio_group ~ kon + age, data = data_full,
##     Hess = T)
##
## Coefficients:
##        Value Std. Error t value
## konM 0.04693  0.0223437    2.10
## age  0.01062  0.0004422   24.01
##
## Intercepts:
##                  Value   Std. Error t value
## Referral|Prio 2 -0.0932  0.0319    -2.9192
## Prio 2|Prio 1    1.8040  0.0339    53.2079
##
## Residual Deviance: 59088.79
## AIC: 59096.79
```

```
summary(fit.glm.p1)
```

```
## Generalized linear mixed model fit by maximum likelihood (Laplace
##   Approximation) [glmerMod]
##  Family: binomial  ( logit )
## Formula: I(prio_group == "Prio 1") ~ kon + scale(age) + (1 | UserName)
##    Data: data_full
##
##      AIC      BIC   logLik deviance df.resid
##  30625.1  30658.0 -15308.5  30617.1    27801
##
## Scaled residuals:
##     Min      1Q  Median      3Q     Max
## -0.8378 -0.5935 -0.5228 -0.4014  2.4910
##
## Random effects:
##  Groups   Name        Variance Std.Dev.
##  UserName (Intercept) 0.125    0.3536
## Number of obs: 27805, groups:  UserName, 21
##
```

```
## Fixed effects:
##             Estimate Std. Error z value Pr(>|z|)
## (Intercept) -1.03032    0.08947 -11.516  < 2e-16 ***
## konM         0.07435    0.02809   2.647  0.00812 **
## scale(age)   0.01135    0.01411   0.804  0.42140
## ---
## Signif. codes:  0 '***' 0.001 '**' 0.01 '*' 0.05 '.' 0.1 ' ' 1
##
## Correlation of Fixed Effects:
##            (Intr) konM
## konM       -0.154
## scale(age) -0.005  0.023
```

```r
summary(fit.glm.p12)
```

```
## Generalized linear mixed model fit by maximum likelihood (Laplace
##   Approximation) [glmerMod]
##  Family: binomial  ( logit )
## Formula: I(prio_group %in% c("Prio 1", "Prio 2")) ~ kon + scale(age) +
##     (1 | UserName)
##    Data: data_full
##
##      AIC      BIC   logLik deviance df.resid
##  32717.5  32750.4 -16354.7  32709.5    27801
##
## Scaled residuals:
##     Min      1Q  Median      3Q     Max
## -3.2406 -1.0259  0.5341  0.6737  1.4808
##
## Random effects:
##  Groups   Name        Variance Std.Dev.
##  UserName (Intercept) 0.1614   0.4018
## Number of obs: 27805, groups:  UserName, 21
##
## Fixed effects:
##             Estimate Std. Error z value Pr(>|z|)
## (Intercept)  0.95885    0.10068   9.524   <2e-16 ***
## konM         0.04119    0.02681   1.536    0.124
## scale(age)   0.46008    0.01319  34.893   <2e-16 ***
## ---
## Signif. codes:  0 '***' 0.001 '**' 0.01 '*' 0.05 '.' 0.1 ' ' 1
##
## Correlation of Fixed Effects:
##            (Intr) konM
## konM       -0.127
## scale(age)  0.020  0.021
```

We find Substantially different coefficients across priority levels, making the interpretation of ordinal models difficult. As such, we report results from binomial models investigating effects regarding the likelihood of any ambulance dispatch and priority 1 ambulance dispatch separately.

Reported model coefficients

```r
cbind(
cbind(est = exp(fixef(fit.glm.p1)),
```

```r
exp(confint(fit.glm.p1,
        method = "Wald")[-1,])),

cbind(est = exp(fixef(fit.glm.p12)),
exp(confint(fit.glm.p12,
        method = "Wald")[-1,]))) %>%
  kable(digits = 3)
```

	est	2.5 %	97.5 %	est	2.5 %	97.5 %
(Intercept)	0.357	0.299	0.425	2.609	2.142	3.178
konM	1.077	1.019	1.138	1.042	0.989	1.098
scale(age)	1.011	0.984	1.040	1.584	1.544	1.626
